# Testing the niche reduction hypothesis for a fossorial rodent (*Geomys bursarius*) experiencing agricultural intensification

**DOI:** 10.1002/ece3.9559

**Published:** 2022-12-12

**Authors:** Nathan Alexander, Bradley J. Cosentino, Robert L. Schooley

**Affiliations:** ^1^ Department of Natural Resources and Environmental Sciences University of Illinois at Urbana‐Champaign Urbana Illinois USA; ^2^ Department of Biology Hobart & William Smith Colleges Geneva New York USA

**Keywords:** agricultural intensification, *Geomys bursarius*, niche hypervolume, niche reduction hypothesis, plains pocket gopher, species distribution model

## Abstract

Habitat loss and fragmentation from conversion to agriculture are known threats to grassland species. However, continued agricultural intensification may further reduce a species distribution and realized niche. Here, we create species distribution models (SDMs) for the plains pocket gopher (*Geomys bursarius*), an ecosystem engineer in grasslands, for historic and contemporary eras in a dynamic agroecosystem and test the “niche reduction hypothesis.” We compare SDMs created from gopher occurrences from the historic era (~1950s, pre‐agricultural intensification) and the contemporary era (post‐agricultural intensification) and assess model transferability. We evaluate shifts in environmental relationships, changes in limiting factors, and an overall decline in niche hypervolume. SDMs were nontransferable between the historic and contemporary eras. Environmental drivers of gopher distribution shifted from elevation, precipitation, and land cover in the 1950s to land cover, soil texture, and soil drainage presently. There also were shifts in environmental associations with gophers now occurring at lower elevations, in sandier soils, and less often in agriculture. Dominant limiting factors of gophers shifted from precipitation to land cover. Gophers were not detected at historic locations during recent resurveys. Contemporary niche hypervolume was reduced compared with the historic niche hypervolume. We found support for the niche reduction hypothesis for a fossorial, grassland species. Further application of the niche reduction hypothesis in landscapes experiencing agricultural intensification is warranted. Understanding niche reduction allows for conservation efforts that promote continued persistence in the contemporary niche while also identifying areas to restore within the historic niche.

## Graphical abstract

Since the 1950s, gophers (*Geomys bursarius*) have experienced a niche shift and a niche reduction in Illinois, concurrent with agricultural intensification. Here, we show that agricultural intensification can continue to reduce a species' niche on landscapes already converted to agriculture.

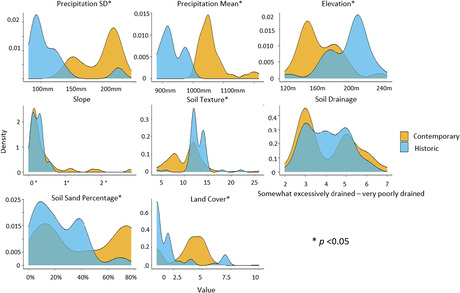

## 1 INTRODUCTION

Habitat loss and fragmentation remain leading threats to biodiversity (Elith & Leathwick, 2009; Haddad et al., 2015) as they often decrease population size (Bartlett et al., 2016), genetic diversity (Lino et al., 2019), and area occupied (Quaglietta et al., 2018; Stewart et al., 2017). However, directly linking habitat change to a species' niche is relatively recent (Scheele et al., 2017). The n‐dimensional hypervolume concept describes how multiple environmental factors constrain a species distribution (Hutchinson, 1957). There are two primary aspects of the niche: the fundamental niche representing the theoretical niche without limitations to environmental resources, and the realized niche representing a subset of the fundamental niche that incorporates limitations such as interspecific competition and dispersal (Hutchinson, 1957; Pulliam, 2000). A species can persist at any combination of environmental conditions within the realized niche hypervolume and, if there were no constraints, within the fundamental niche hypervolume. Considering the niche as a hypervolume has expanded niche‐based research to include variation among populations, connections between niche variation and genetic variation (Holt, 2009), differing responses of life stages to climate variability (Jackson et al., 2009), and stronger links between niche hypervolume and geographic space (Colwell & Rangel, 2009; Soberon & Nakamura, 2009). Continued application of the niche concept has recently resulted in the “niche reduction hypothesis” (Scheele et al., 2017).

The niche reduction hypothesis highlights that novel threats to a species may not be uniform across the species' realized niche (sensu Hutchinson, 1957), thus leading to a reduced niche hypervolume as well as range reduction (Scheele et al., 2017). For example, invasive cats inhabited less complex habitats of the central rock‐rat (*Zyzomys pedunculatus*), limiting the central rock‐rat's realized niche to more rugged terrain (McDonald et al., 2018). Due to strong predation specifically in less complex habitats, the prey species' environmental niche was reduced, omitting niche spaces that were historically occupied in specific environmental conditions. In addition to introduced predators, novel diseases (Scheele et al., 2019) and land conversion (Rutrough et al., 2019) may have effects on a subset of a species' realized niche.

Understanding niche reduction in agricultural landscapes is a useful next step for global conservation efforts as agriculture is a major driver of landscape change worldwide (Newbold et al., 2015; Ramankutty & Foley, 1998, 1999). Initial conversion of landscapes to agriculture may reduce a species' realized niche (Rutrough et al., 2019), but agricultural expansion and intensification may promote further niche reduction. Agricultural expansion converts high‐quality habitats into crop fields (Lark et al., 2020) including bringing lands into development as they expire from conservation programs (Morefield et al., 2016). Once a landscape is converted to agriculture, agricultural practices evolve to meet increased yield demands (Hunter et al., 2017), and this agricultural intensification can increase soil compression (Keller & Or, 2022) and decrease species richness (Carmona et al., 2020; Raven & Wagner, 2021). Although agricultural intensification produces higher crop yields per area farmed, it does not necessarily prevent agricultural expansion (Rudel et al., 2009), and farmers may prioritize land resilient to flooding for agricultural production (e.g., higher elevations; Yang et al., 2021). Continued agricultural expansion occurs, albeit at a lower rate, in tandem with agricultural intensification (Lin & Huang, 2019).

Despite global effects of agriculture on species, the niche reduction hypothesis has only rarely been applied to agricultural land conversion, and to our knowledge, not to agricultural intensification. In one example involving land conversion, a burrowing mammal's historic range was estimated to be 56% greater than its previously defined historic range, with a shift in the current realized niche due to agricultural development on natural lands (Rutrough et al., 2019). However, it remains unknown whether the niche reduction hypothesis applies more broadly across altered landscapes, especially those experiencing agricultural intensification after most initial land conversion has occurred. If it does, this hypothesis could provide a deeper understanding of niche dynamics and direct land management by identifying drivers of niche reduction or changes in limiting environmental factors.

To further define the scope of the niche reduction hypothesis, we apply the concept to the Midwestern region of the United States, a landscape highly modified by agriculture (Lark et al., 2020; Ramankutty et al., 1999; Ramankutty & Foley, 1998). Specifically, Illinois often leads in agricultural intensifying practices in North America (Warner, 1994). Farmers in Illinois have transitioned native haying grasses to European species and increased the use of annual row crops, synthetic fertilizers, and mechanization (Warner, 1994). Between the 1950s and the 1980s, cash‐crop farms in Illinois increased in individual size with mid‐sized farms growing to ≥400 ha each (Garcia et al., 1987). This increase in farm size is associated with growing corn, expanded soybean production, and a reduction in hay, oats, and pasture (Hart, 1986; Sulc & Tracy, 2007; Warner, 1994). One impact of this agricultural intensification is that fence rows and breaks between crop fields were eliminated (Hart, 1986). These landscape changes occurred heterogeneously across Illinois since the 1950s (Mankin & Warner, 1999; Warner, 1994), possibly due to farmland–soil and crop yield–soil relationships (Garcia‐Paredes et al., 2000; Iverson, 1988; Mause, 1971). Such spatial heterogeneity may promote niche reductions for species associated with grassland habitat.

**FIGURE 1:**
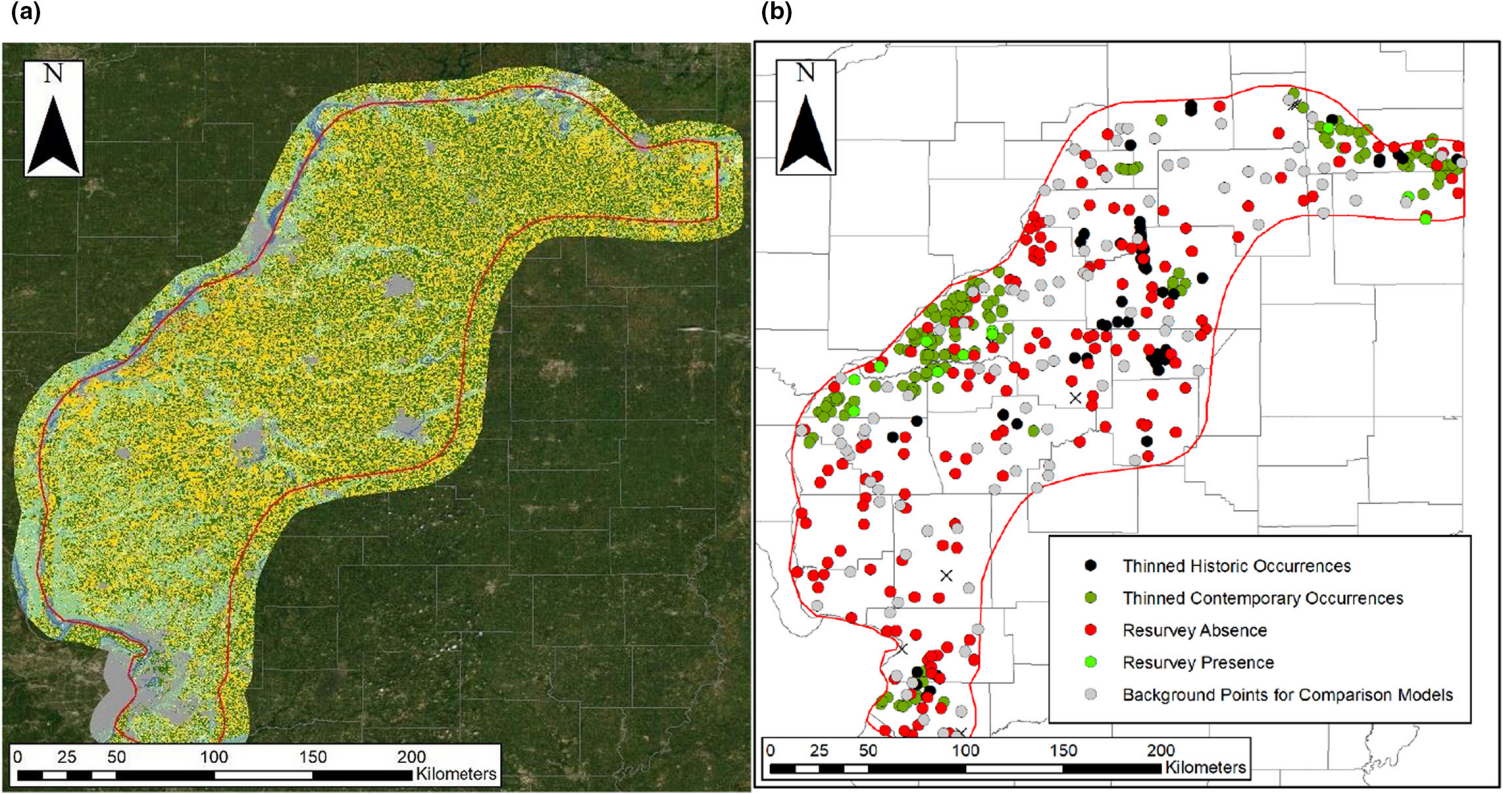
(a) *Geomys bursarius* historic range (red line) in Illinois (Hoffmeister, 1989) buffered by 10 km with contemporary land cover (gray = development, yellow = corn, dark green = soybean, light green = grassland/pasture). (b) Thinned historic gopher occurrences, thinned contemporary gopher occurrences, contemporary model validation absences, contemporary model validation presences, and the 100 background points used for comparative MaxEnt models across the gopher range in Illinois.

Here, we tested for shifts in a species distribution and niche hypervolume between two eras: a historical era post land conversion but before recent agricultural intensification, and a contemporary era reflecting current agricultural practices. We selected a fossorial mammal, the Illinois plains pocket gopher (*Geomys bursarius illinoensis*), as our focal species. It has one of the more restricted ranges of eight subspecies (Figure 1a; Connior, 2011; Hart, 1971) and is the only subspecies that persists east of the Mississippi River (Hart, 1971) and south of the Illinois River (Hoffmeister, 1989). Rivers are often boundaries to distributions and may serve as dispersal barriers due to the poor swimming ability of pocket gophers (Kennerly, 1963; Komarek & Spencer, 1931). The distribution of *G. bursarius* in Illinois has not been studied since 1935 (Mohr, 1935), and its environmental niche has never been estimated. Gophers also are ecosystem engineers that contribute greatly to ecosystem processes by altering vegetation composition and biomass and soil properties (Huntly & Reichman, 1994; Reichman & Seabloom, 2002; Romañach et al., 2005). Therefore, changes in the area occupied or realized niche of gophers will affect functioning of agroecosystems.

Historically, gophers inhabited tallgrass prairie, but now also inhabit other grasslands, some cultivated fields, and urban areas. Native prairies in Illinois have drastically declined with only 0.01% remaining (Robertson et al., 1997). From 1820 to 1978, there was a reduction from 8.76 million ha to 931 ha, with most prairies converted to agriculture (Iverson, 1988). Most (83%) remaining prairies are <4 ha, and 30% are <0.4 ha (Klopatek et al., 1979), creating a highly fragmented landscape for a species historically associated with prairies. Agricultural conversion and intensification may remove other grassy refuges for burrowing mammals such as bringing road rights‐of‐way into production (Martin & Heske, 2004). Thus, we would expect a niche reduction if specific land cover types within the gopher's historic niche were disproportionately impacted by agricultural expansion and intensification.

Second, gophers have strong soil texture associations, yet they can occur in a range of soil textures (Connior, 2011) including silt loam soils with <1% sand to sandy loam soils (Komarek & Spencer, 1931). Species distributions of gophers are related to soil sand and loam percentages likely due to burrow construction and friability; however, these associations range drastically between species (Connior, 2011). Illinois had prairies in sand, black soil, gravel, and other soil categories (Robertson et al., 1997; White, 1978), potentially leading to both landscape and soil associations for gophers. If prairies in specific soil types were lost to agriculture, gophers may exhibit two distinct responses: maintain associations with soils regardless of land cover or become extirpated from specific soils due to land cover change. Agricultural intensification may also occur on specific soils as farming practices or crop types changed. Hence, we may expect a niche reduction if agricultural intensification impacted soil types nonuniformly.

Third, precipitation in Illinois has increased over time with a 10‐year moving average of annual precipitation rising from ~920 mm in 1960 to ~1020 mm in 2011. Extreme precipitation events are also increasing (Dahal et al., 2018). Heavy rains may cause pooling in poorly drained soils (Krichels et al., 2019), leading to flooding of gopher burrows and mortality (Connior & Risch, 2010). Thus, changing climate may affect species distributions and realized niches (Wiens et al., 2009).

To address our overarching hypothesis that gophers in Illinois experienced a niche reduction due to agricultural intensification since the 1950s, we developed species distribution models (SDMs) for current and historical eras to identify limiting environmental factors. Next, we created comparable models for the two eras to quantify changes in distribution and environmental associations. Finally, we estimated Hutchinsonian niche hypervolumes to determine whether there was a reduction in the gopher's realized niche over time. We predicted that both the contemporary and historic niches for gophers would include a preference for grasslands, sandy‐clay loam soils, higher elevations, and sites with lower precipitation and better soil drainage. We expected a decline in the suitability of agricultural land cover since the 1950s due to intensification and a shift to cultivated annual crops. Although grasslands are hypothesized to be the most suitable habitat even contemporarily for gophers, we expected grassland suitability to decrease over time due to continued small‐scale land conversion, leaving grasslands in poorer soils and lower productivity areas. Collectively, these changes should result in a narrower niche hypervolume for gophers for the contemporary era relative to the historic era.

## 2 METHODS

### 2.1 Contemporary SDM

We created a contemporary SDM (2008–2019) for pocket gophers in Illinois to assess the current realized niche. SDMs use occurrence records to predict the distribution of species using environmental variables. Presence–pseudoabsence SDM models have increased in use, allowing collation of multiple sources of occurrence records (Valavi et al., 2021). Here, we combined gopher presences (*n* = 204 total, Figure 1b) from four sources: (1) a presence–absence survey developed with stratified sampling (*n* = 12), (2) a presence‐only driving survey (*n* = 142), (3) downloaded presences from the Global Biodiversity Information Facility from 2008 to 2019 (*n* = 23), and (4) live‐trapping of gophers (*n* = 27; University of Illinois Urbana‐Champaign Institutional Animal Care and Use Committee protocol #17190). For the live‐trapping, we followed Sikes and The Animal Use and Care Committee of the American Society of Mammalogists (2016) ethical guidelines using both box traps (Connior & Risch, 2009) and bucket traps (Moore et al., 2019). Traps were checked every 3 h. Initially, we conducted a presence–absence survey across the historic range in 2016–2017 and surveyed 75 grassland sites using both walking transects (2–4 transects of 100 m each within grasslands, including linear configurations such as roadside berms) and time‐limited searches (15 min). We determined current gopher presences by identifying active gopher mounds (Quinn et al., 2010). Because of low occurrence rates, we shifted to more extensive presence‐only surveys in which we drove across much of the historic range (≥2750 km) in 2019 and again identified gopher presences from active mounds.

MaxEnt (Phillips et al., 2006; Phillips et al., 2017) estimates the relative probability of occurrence by comparing environmental covariates at occurrences to those at background points (Elith et al., 2011). This allows complex transformations to estimate relative habitat suitability and then predict suitability across the study region (Elith et al., 2011). We only modeled within the historic range of gophers in Illinois, with a 10 km buffer, refraining from projecting any models outside of the study area. We retained presences >1 km apart (*n* = 150) using the “spThin” package (Aiello‐Lammens et al., 2015) in R (R Core Team v. 4.1.1) because *G. bursarius* generally have home ranges <0.5 km^2^ (Connior & Risch, 2010; Zinnel, 1992).

**FIGURE B1:**
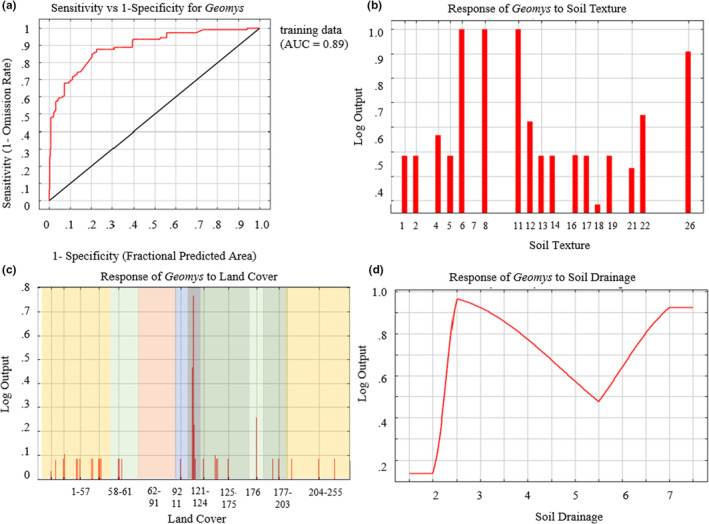
(a) Area under the curve and (b–d) response curves where Log Output represents the change in occurrence probability as the environmental parameter is varied for (b) soil texture, (c) land cover, and (d) soil drainage for the top contemporary MaxEnt model for pocket gophers in Illinois. (b) Gophers were more likely to occur in silty clay loam (6), sandy loam (8), stratified sand to loam (11), and variable soils (26). (c) Gophers were more likely to occur in low‐development areas (121), and not occur in agriculture (yellow), grasslands or pastures (light green), orchards (red), water and wetlands (blue), developed areas (grey), and forests (dark green). (d) Gophers were also likely to occur in very poorly drained (7) and well‐drained (3) soils. For full numeric associations of soil texture, land cover, and soil drainage, refer to Tables A1, A2, A3 in Appendix A.

**FIGURE B2:**
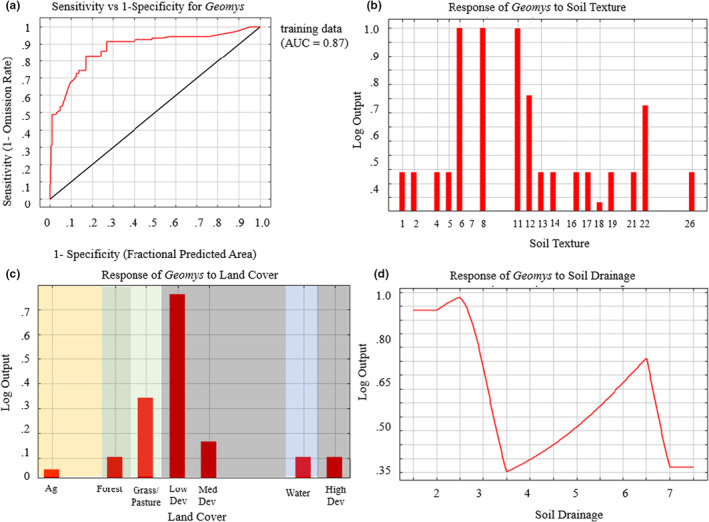
(a) Area under the curve and (b–d) response curves for the selected model using fewer land cover classifications. Log Output represents the change in occurrence probability as the environmental parameter is varied for (b) soil texture, (c) land cover, and (d) soil drainage for the top contemporary MaxEnt model for pocket gophers in Illinois. (b) Gophers were more likely to occur in silty clay loam (6), sandy loam (8), stratified sand to loam (11), and variable soils (26). (c) Gophers were more likely to occur in low‐development areas, and not occur in agriculture (yellow), forests (dark green), grasslands or pastures (light green), water and wetlands (blue), and medium and highly developed areas (grey). (d) Gophers were also likely to occur in very poorly drained (1) and excessively drained (6) soils. For full numeric associations of soil texture, land cover, and soil drainage, refer to Tables A1, A2, A3 in Appendix A.

We created a suite of environmental models at an ~30 m × 30 m resolution incorporating land cover (USDA National Agricultural Statistics Service, 2019), soil texture categories, soil drainage classes, percent sand (Soil Survey Staff, 2018), elevation (U. S. Geological Survey, 2006), and precipitation mean and standard deviation from 2008 to 2018 (PRISM Climate Group, 2014). Land cover included crop types (*n* = 39), grasslands and pasture types (*n* = 7) development intensity (*n* = 3), forest types (*n* = 6), water (*n* = 1), and orchards (*n* = 7; Table A3 in Appendix A). To ensure that we did not overparameterize the model, we also assessed SDMs using a reduced land cover classification (*n* = 7; Figures B1 and B2 in Appendix B). Soil texture, drainage class, and percent sand spatially varied predominantly based on past glaciation (Piskin & Bergstorm, 1975) with 16 categories for soil texture, six categories of drainage ranging from poorly drained to excessively drained (Tables A1 and A2 in Appendix A), and sand percent ranging from 0% to 90%. These environmental variables were rasterized to match the resolution of the elevation raster. There was limited variation in elevation, ranging from 112 to 281 m with slopes ranging from 0 to 27.5°. Rainfall data were resampled from an 800 m × 800 m grain to ~30 m × 30 m using the nearest neighbor method. We averaged annual mean precipitation over 10 years (2008–2018), with yearly mean precipitation ranging from 899 to 1264 mm (SD = 103–292 mm). There was higher mean precipitation in the south and higher deviation in precipitation toward the center of the gopher range. All eight layers had a variance inflation factor (VIF) <2, and they were not strongly correlated (*r* < .6) and well within acceptable levels of parameter correlation for MaxEnt (Syfert et al., 2013). We further tested multicollinearity of the land cover and soil texture layers by extracting 1000 random cell values and conducting chi‐square tests between all the variables (all *p* > .25).

We ran MaxEnt v3.4.1 across all combinations of the eight environmental layers and two values of smoothing parameters (*β* = 1 or 2) with 10,000 background points and 75% of the thinned occurrence data (*n* = 106), leaving 25% for model testing. We selected the top models using Akaike's Information Criterion corrected for small sample size (AIC_c_) in ENMTools (Warren et al., 2010) and then calculated area under the curve (AUC) for the training and test data. We also performed a Jackknife analysis to measure the relative contribution of each environmental variable in the model (Elith et al., 2011).

In addition to model testing using 25% of the data, we conducted a field validation of our top model during June–July 2020. We binned predicted habitat suitability into four classes (Rhoden et al., 2017) and sampled >20 points in each class: 0–0.059 (Very Low, *n* = 48; up to the lowest 5% of estimated habitat suitability at gopher occurrences from the contemporary model), 0.06–0.49 (Low, *n* = 38), 0.5–0.749 (Good, *n* = 23), and 0.75–1 (Very Good, *n* = 64). During the initial field validation, we noticed most “Very Good” sites were within parks or lawns within cities and towns, so we included additional randomly selected sites with “Very Good” habitat suitability that were outside of cities or towns (*n* = 34). To ensure property access, we restricted all validation sites to be near roads. Overall, we surveyed 173 new sites (Figure 1b).

We then used a random forest classification tree to explain why gophers occurred only at a subset of suitable areas or occurred in areas with low estimated habitat suitability (Egly & Larson, 2018). We modeled gopher occurrences from the field validation survey as a response to observed land cover recorded during validation surveys and distance to known gopher occurrence. We included observed land cover in case there was disagreement between observed land cover and the land cover raster layer. We did not include variables used in the MaxEnt model in the random forests to prevent circularity. We assessed classification accuracy by estimating the difference between the out‐of‐the‐bag error rate and parameter permutation across all trees and then normalized by the SD between runs. We estimated node impurity through the Gini index to assess parameter importance (a low Gini index indicates high parameter importance) using the varImpPlot function in the “RandomForest” R package (Breiman, 2001).

### 2.2 Historic SDM

We next created a historic SDM using occurrence records from 1945 to 1955, a period ~45 years after most of the land conversion in Illinois (Robertson et al., 1997). We obtained historic occurrences of *G. bursarius illinoensis* from museum specimens from the Illinois Natural History Survey (Champaign, IL; *n* = 78) and the Field Museum of Natural History (Chicago, IL; *n* = 6). These occurrences were then combined and thinned to be ≥1 km apart (*n* = 56, Figure 1b). Due to limited sample size, we did not withhold 25% of the data for model testing.

For historic land cover, we georeferenced aerial imagery from 1945 to 1955 (USDA & USGS, 2020) of counties within *G. bursarius illinoensis*' range using the georeference tool in ArcMap (Esri ArcMap v. 10.7.12019) by creating control points between the digital image and ArcMap satellite base imagery (Esri Digital Globe 2019) at stationary locations (e.g., county borders and road or river features). We georeferenced 105 aerial images using an average of 41 control points (SD = 34) and a root mean square error of 102 m (SD = 69 m). We then buffered the thinned historic occurrences by 1 km and digitized the land cover into nine categories: Agriculture, Agricultural Berm, Forest, Grassland or Pasture, Mild Development, Medium Development, Roads, Water, and Unknown. All the categories were represented in the contemporary model except for agricultural berm and roads. The reduction in land cover classes from the contemporary model was necessitated by our inability to identify crop types or forest types from aerial photography (see Historic vs. Contemporary Models section for comparison models). Mild Development differed from Medium Development by density of buildings. Mild Development could include stand‐alone houses or structures in farmland or woods, and Medium Development generally included towns or neighborhoods. We defined Agricultural Berm as a division between two agricultural fields that was not along a road to highlight potential grassland refugia for *G. bursarius illinoensis*. We used the same raster layers as for the contemporary SDM for soil texture, drainage class, percent sand, elevation, and slope, and we used the 10‐year precipitation mean and SD from 1945 to 1955 (PRISM Climate Group, 2014). However, the 10‐year precipitation SD had a VIF = 2.7, so models either included mean precipitation or precipitation SD, but not both.

Like the contemporary SDM, we ran MaxEnt models across all combinations of parameters and two smoothing parameters (*β* = 1 or 2). We were only able to generate 100 background points ≥1 km apart within the historic *G. bursarius illinoensis*' range because we did not have a range‐wide land cover classification and were constrained by our digitization capacity. We hand‐digitized land cover from aerial photographs within 1 km buffers around these 100 background points to use in MaxEnt. We selected the top models using AIC_c_ in ENMTools. We also performed a Jackknife analysis to identify parameter contributions of the top models.

### 2.3 Historic versus contemporary SDMs

Species distribution models are viewed as temporally static realized niches (Guisan & Theurillat, 2000). The niche reduction hypothesis expands the connection between SDMs and the realized niche by incorporating temporal dynamics as well as novel threats to a species. To create comparable models to examine changes in gopher niches between eras, we used the global models with all environmental predictors (hereafter “full models”) of the contemporary and historic SDMs (precipitation SD included despite the VIF > 2). We reclassified the contemporary land cover to the nine land cover types used for the historic SDM (Table A3 in Appendix A). We then re‐ran the full models using the same 100 background points for both the contemporary and historic models and compared parameter contributions with Jackknife analyses. We further compared models by estimating niche overlap between the contemporary and historic SDMs. We used Warren's *I* index, an estimation of niche similarity where 0 indicates no overlap and 1 indicates perfect overlap (Warren et al., 2008), in ENMTools. The niche overlap estimates the spatial overlap of SDM raster layers, providing a spatial estimate of niche similarity, but does not account for variation in contributing parameters. We also used Warren's *I* to compare model performance.

To determine specific niche shifts and possible reductions, we compared contemporary and historic gopher occurrences using Wilcoxon rank‐sum tests for continuous environmental variables and Pearson's *χ* ^2^ Tests for categorical variables (soil texture and land cover). If there was a loss of specific niche space, a niche reduction would be supported. However, niche shifts could also occur in which the overall niche volume is constant between eras, but occurrences have shifted to a different predominant environmental space (e.g., gophers occur across a similar range of soil sand percentages, but currently select sandier soils than historically). We also used the “Limiting Factor” analysis in MaxEnt to determine which parameters were limiting at gopher occurrences and broadly across the predicted areas (Elith et al., 2010).

Although creating comparable models is necessary to determine a niche shift, we also wanted to assess model transferability and niche overlap for the best models selected from our initial contemporary SDM and historic SDM (see above). We extracted the predicted habitat suitability for historic occurrences, contemporary occurrences, and background points for each top MaxEnt model. If the contemporary model predicted high suitability for historic occurrences, and the historic model predicted high suitability for contemporary occurrences, there would be temporal stability of the SDMs. For the contemporary model, we also extracted habitat values from absences (*n* = 47) from the presence–absence survey conducted in 2017 to determine whether the model predicted known absences to be low habitat suitability. We also visited locations where gopher specimens were collected between 1945 and 1981 to determine persistence and contemporary land cover. We confirmed active gopher mounds on foot except along interstates.

### 2.4 Niche hypervolumes

Although the above methods can identify niche shifts related to environmental parameters, we also estimated niche hypervolumes to determine directly whether there was a reduction in volume between the two eras. Because nonordinated categorical variables (e.g., soil texture and land cover) cannot be directly incorporated into hypervolume calculations (Blonder et al., 2018), we ordinated our data. First, we scaled and centered continuous parameters by subtracting the parameter means from the values and then dividing by the parameter SDs. Next, we used PCAMix from the R package “PCAmixdata” (Chavent et al., 2017) for ordination, which allows for mixed data and categorical parameter interpretation (Carvalho & Cardoso, 2020). This method integrates principal component analysis with multiple correspondence analysis.

Finally, we calculated the hypervolume using a Gaussian kernel density estimate (KDE) of the first 4 axes to ensure appropriate power given the number of observations (Blonder et al., 2017; Mammola & Cardoso, 2020) using the “Hypervolume” R package (Blonder et al., 2018). Although one‐class support vector machine (SVM) estimation also is appropriate for calculating realized niche volume (Blonder et al., 2018; von Takach et al., 2020), we opted for Gaussian KDE methods that may better identify holes (Mammola & Cardoso, 2020). Given that our data included transformed categorical variables, and our predicted niche loss may be within the center of the niche hypervolume, the tight, binary wrap of SVM (Blonder et al., 2018) may overpredict niche volume for our dataset. Gaussian KDEs have produced appropriate hypervolume estimates for SDMs and for the realized niche (Blonder et al., 2017). We used a kernel bandwidth based on the Silverman estimator (Silverman, 1998). We also report the centroid distance between the two hypervolumes, and the Sørensen similarity to quantify overlap, following recommended best practices (Lu et al., 2021; Mammola & Cardoso, 2020).

## 3 RESULTS

### 3.1 Contemporary SDM

**FIGURE 2:**
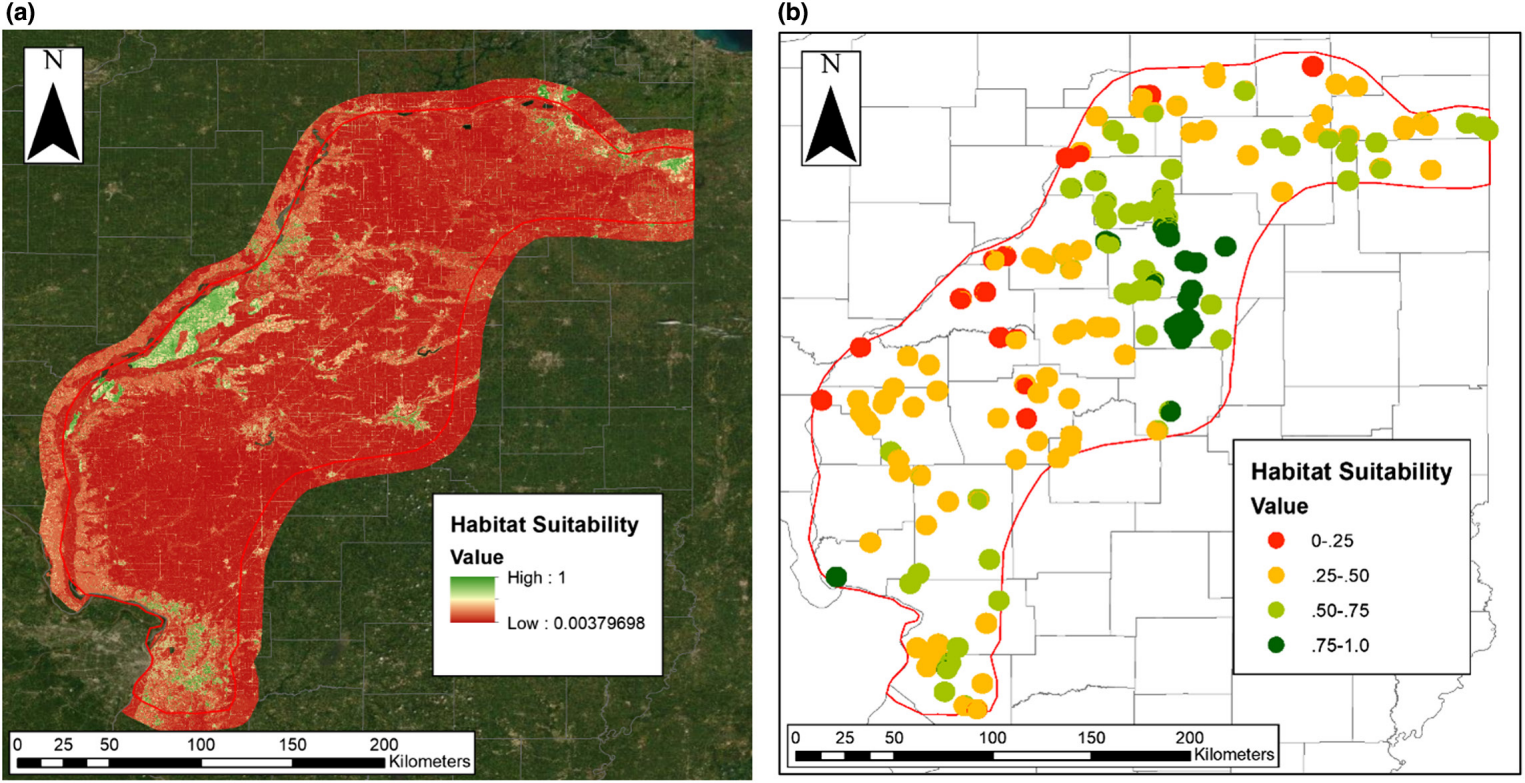
(a) Habitat suitability from the top contemporary species distribution model for pocket gophers across the historic range in Illinois (buffered by 10 km). Parameters included land cover, soil texture, and soil drainage. (b) Habitat suitability from the top historic species distribution model for pocket gophers across the historic range (red line) in Illinois. Parameters included elevation, mean precipitation, and land cover.

**FIGURE C1:**
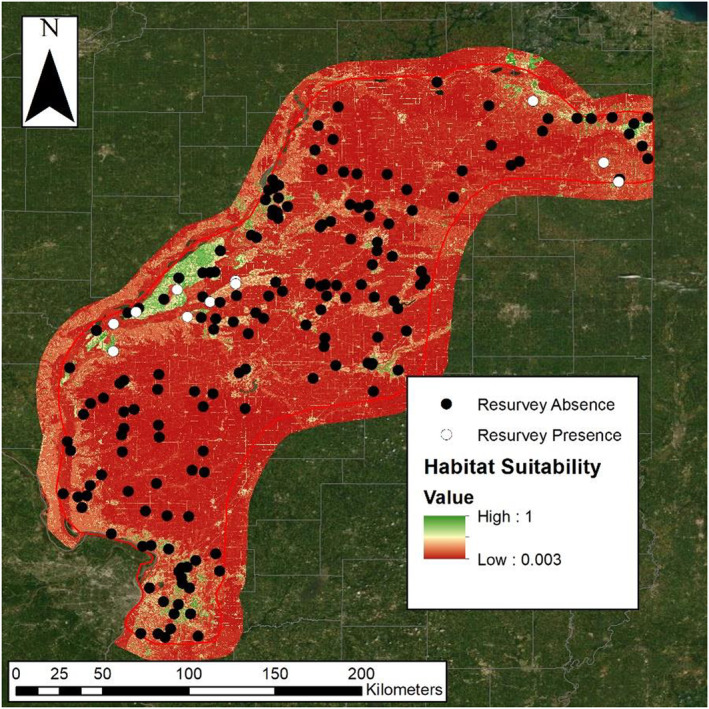
Validation survey presences (white) and absences (black) across the range and predicted contemporary habitat suitability of *Geomys bursarius illinoensis*.

The top contemporary MaxEnt model (Figure 2a) indicated the gopher's current distribution is best predicted by land cover, soil texture, and soil drainage (AUC_training_ = 0.89). Gophers selected for mildly developed spaces; silty‐clay loam, sandy loam, and stratified sand to loam soils; and areas with high drainage capacity (Figure B1 in Appendix B). Land cover contributed the most to the model, followed by soil texture, as determined by the jackknife analysis (Figure B1 in Appendix B). Agricultural land cover classes all had <0.12 probability of suitable conditions based on the response curves (Figure B1 in Appendix B). The contemporary model fit the 25% testing data well (AUC_testing_ = 0.90). Moreover, there was no evidence that the model was overparameterized (Figures B1 and B2 in Appendix B). The alternative model with a reduced number of land cover categories produced similar results, including the same predictors with similar response curves and jackknife analysis, and had a high spatial niche overlap (Warren's *I* = 0.99) with the selected contemporary model (Figures B1 and B2 in Appendix B; Figure C1 in Appendix C).

**FIGURE C2:**
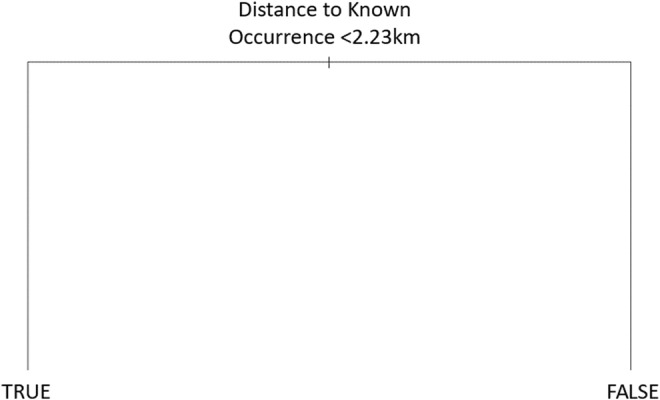
Classification tree for field validation points. Positive detections were within 2.2 km of a previously identified occurrence, and observed land cover did not contribute to occurrence probability.

**FIGURE C3:**
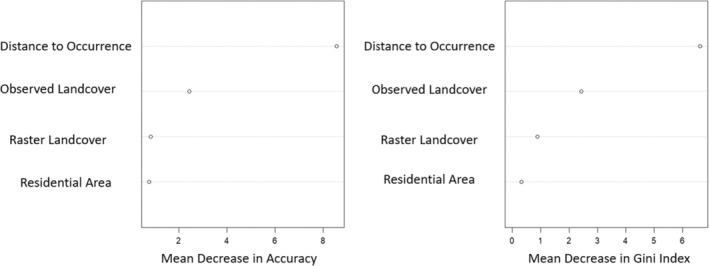
Results of random forest plots from field validation surveys for pocket gophers in Illinois. The variable “distance to previously known occurrence” had the greatest impact on accuracy and the Gini index.

During field validation, we found eight novel gopher occurrences in “Very Good” habitat, one in “Good” habitat, and one in “Low” habitat (Figure 1b). We did not find any gophers in habitat with a “Very Low” suitability classification. Gophers occurred in grassland/pastures (*n* = 2), fallow fields (*n* = 1), developed/open spaces (*n* = 2), and grass berms surrounded by corn (*n* = 3) or soybeans (*n* = 3). The gopher occurrences in “Good” and “Low” habitat had a misclassification of land cover in which the observed land cover was “fallow field” and “grassland/pasture,” whereas the raster indicated “Developed/Low Intensity” and “Developed/Open Space,” respectfully. All new gopher occurrences were within 2.23 km of a known gopher location. For modeling with the contemporary land cover (raster resolution of 30 m × 30 m), small grass berms were often aggregated with the surrounding land cover, possibly leading the selected contemporary MaxEnt model to underpredict occupancy in these sites. For the classification tree, the residual mean deviance was 0.42 with a misclassification rate of 0.063 (Figures C2 and C3 in Appendix C).

### 3.2 Historic SDM

**FIGURE B3:**
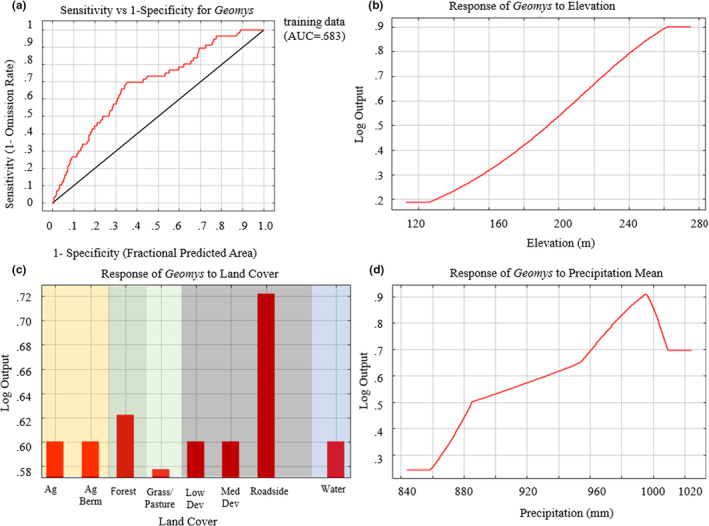
(a) Area under the curve and (b–d) response curves where Log Output represents the change in occurrence probability as the environmental parameter is varied for (b) elevation, (c) land cover, and (d) precipitation mean for the top historic MaxEnt model for pocket gophers in Illinois. (b) Gophers were more likely to occur at higher elevation (260 m), (c) along roadways compared with agriculture and agriculture berms (yellow), forests (dark green), grasslands or pastures (light green), low or medium development (grey), or water and wetlands (blue), and (d) in areas with ~980 mm of rainfall.

**FIGURE B4:**
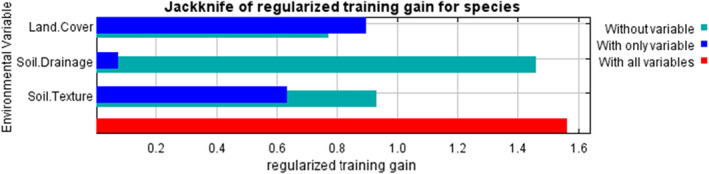
Jackknife analysis of parameter contribution of the top contemporary habitat suitability model for pocket gophers in Illinois. Land cover contributed the most to the regularized training gain and soil drainage contributed the least.

**FIGURE B5:**
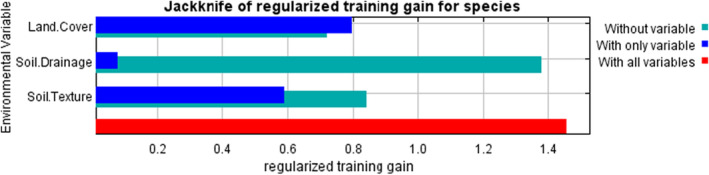
Jackknife analysis of parameter contribution of the top contemporary habitat suitability model for pocket gophers in Illinois with reduced land cover classification. Land cover contributed the most to the regularized training gain and soil drainage contributed the least.

**FIGURE B6:**
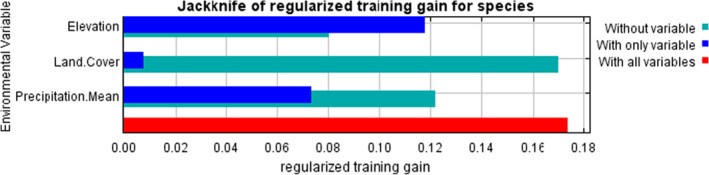
Jackknife analysis of parameter contribution of the top historic habitat suitability model for pocket gophers in Illinois. Elevation contributed the most to the regularized training gain and land cover contributed the least.

The top historic MaxEnt model (Figure 2b) indicated the gopher's historic distribution is best predicted by elevation, mean precipitation, and land cover (AUC_training_ = 0.68, Figure B3 in Appendix B). Gophers selected for areas with higher elevation, higher precipitation, and along roadsides. Elevation contributed the most to the model, followed by precipitation (Appendix B). However, unlike the contemporary model, agricultural land cover and agriculture berms both had a probable suitability of 0.60 predicted by the response curves (Figure B3 in Appendix B). All agricultural land cover was aggregated in these models, so there is limited inference on which types of crops may be more suitable (Figures B4, B5, B6 in Appendix B).

### 3.3 Historic versus contemporary SDMs

**FIGURE 3:**
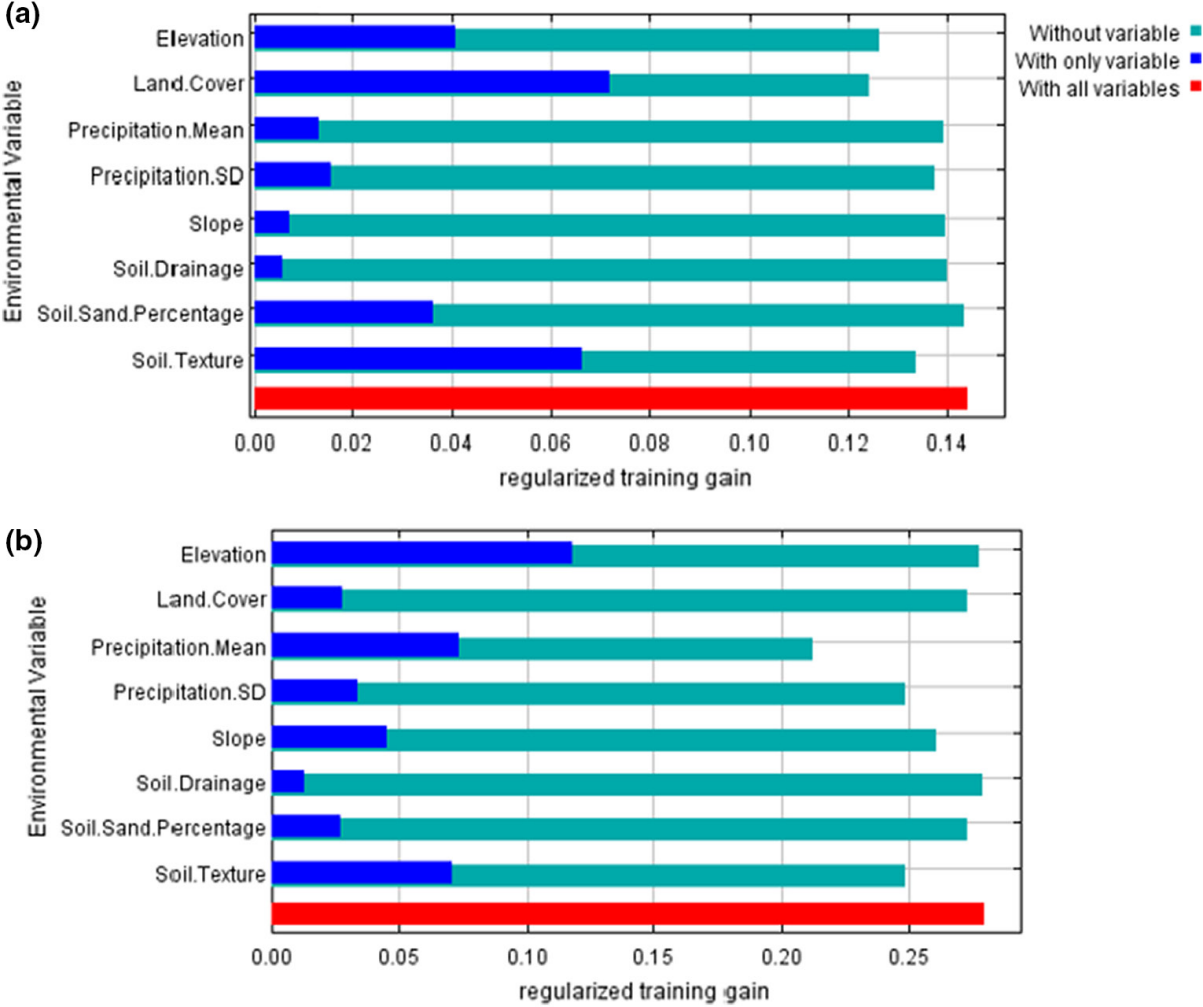
Jackknife analysis of parameter contribution representing the gain when a parameter is used in isolation (blue), the decrease in gain when the parameter is omitted (teal), and the gain when all the parameters are included (red) from the comparable (a) contemporary species distribution model, and (b) historic species distribution model for pocket gophers in Illinois.

The historic and contemporary SDMs had different limiting factors, did not spatially predict similar habitat suitability, and had shifted environmental associations at gopher occurrences. Elevation and precipitation contributed the most to the historic full model (AUC_training_ = 0.75), whereas soil texture, land cover, and sand percentage contributed the most to the contemporary full model (AUC_training_ = 0.67 with 100 background points; Figure 3). The historic and contemporary full models, when restricted to the same 100 background points, had a spatial niche overlap (Warren's *I*) of 0.92. The best contemporary model and the best historic model had a Warren's *I* of 0.79 (Appendix D). The contemporary full model with 10,000 background points and with 100 background points produced similar models, although there was a decrease in AUC that is expected with lower statistical power. Furthermore, the distance between occurrence records and background points had a similar distribution between the two eras, leading us to conclude that our models are comparable (Table D1 in Appendix D).

**FIGURE 4:**
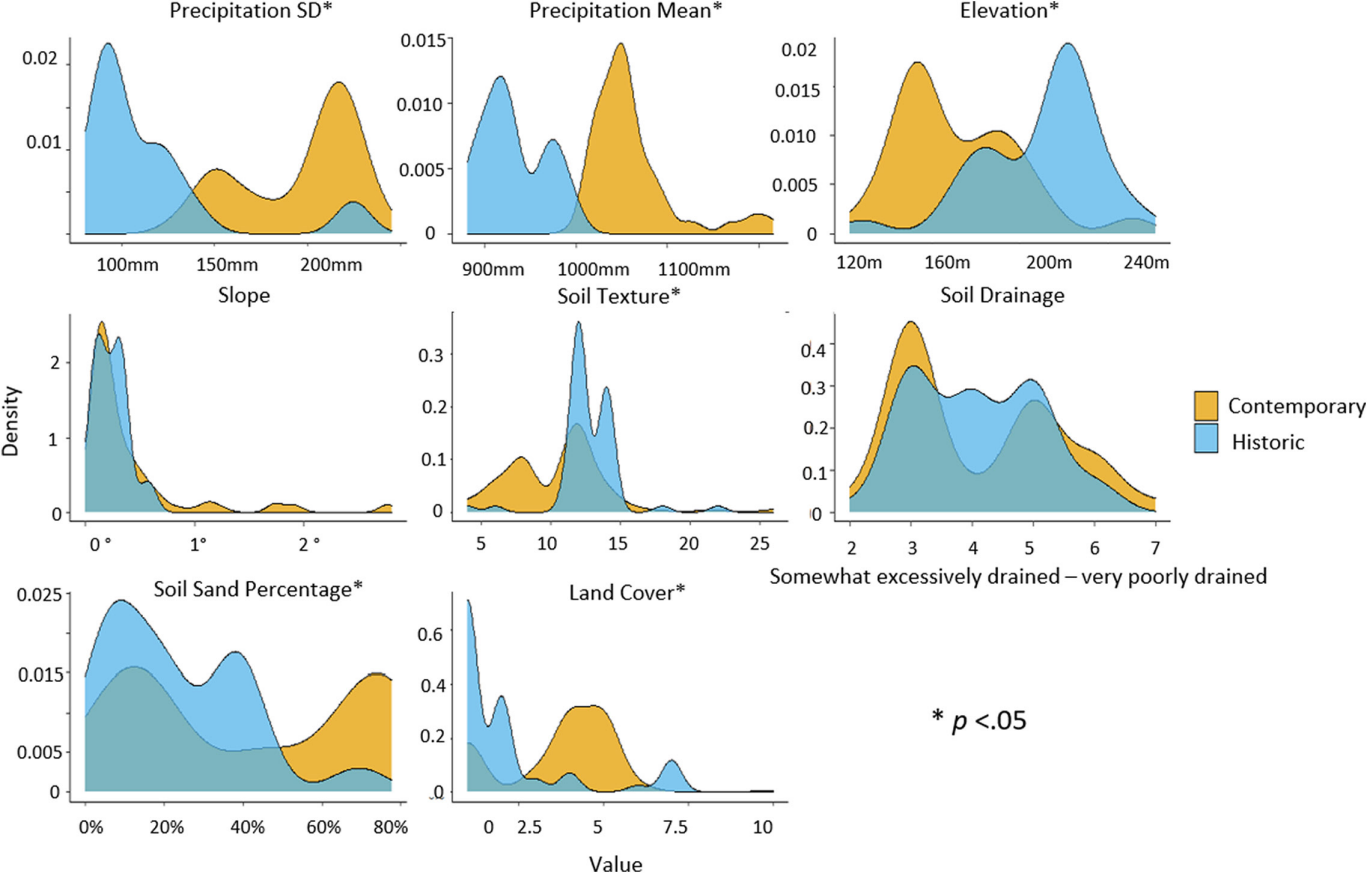
Comparison of environmental parameter values at historic and contemporary occurrences for pocket gophers in Illinois. Soil texture and land cover are categorical variables (Table A3 in Appendix A). Significant niche shifts are indicated based on Wilcoxon rank‐sum tests for all parameters except land cover and soil texture, which were evaluated with Pearson's *χ* ^2^ tests. Gophers now occur at areas with ~1050 mm (SD = ±150 mm) of precipitation, elevations at ~150 m, in silty clay and sandy loam soil texture; well‐drained and somewhat poorly drained soils; and soils with low and high sand percentage. Gophers also occur in mildly developed areas. Historically, gophers occurred at locations with ~910 mm (SD = ±90 mm) of precipitation; elevations at ~220 m; silty clay and very cobbly silt loam soil texture; well‐drained, moderately well‐drained, and somewhat poorly drained soils; soils <40% sand percentage; and predominantly in agriculture and along agricultural berms. Although there were niche shifts of most environmental parameters from historic to contemporary occurrences, there was no evidence for a niche shift for slope (*p* = .34) or soil drainage (*p* = .71).

**FIGURE C4:**
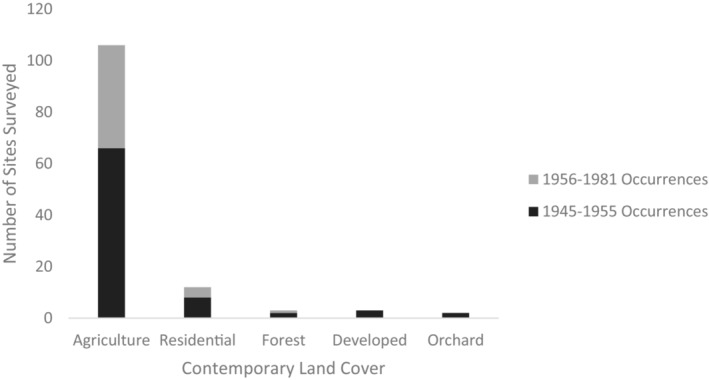
Contemporary land cover at historic occurrences of pocket gophers in Illinois from 1945 to 1955 (black) and 1956 to 1981 (grey). No individuals were detected at the locations of the historic occurrences during contemporary resurveys.

**FIGURE E1:**
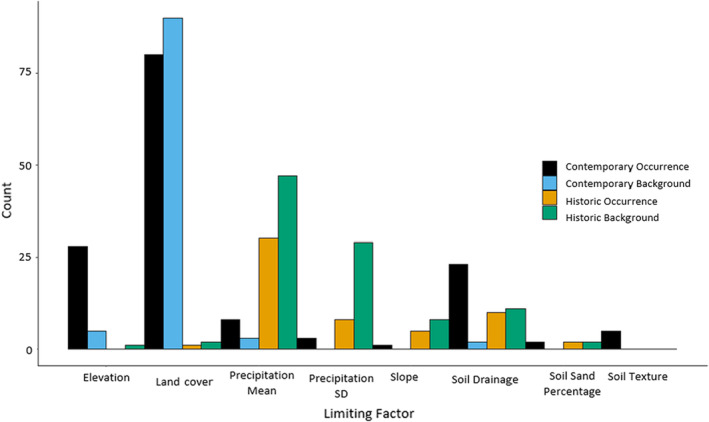
Limiting factors for the full contemporary MaxEnt model at contemporary gopher occurrences (black) and 100 background points (blue) and limiting factors for the full historic MaxEnt model at historic gopher occurrences (yellow) and 100 background points (green).

**FIGURE E2:**
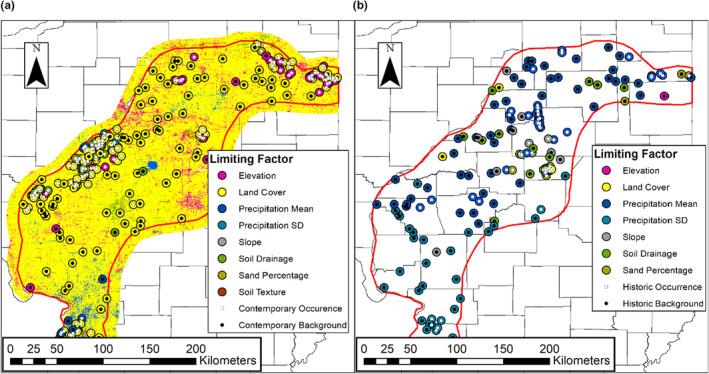
*Geomys bursarius* limiting factor maps for (a) the full contemporary MaxEnt model with 10,000 background points and (b) the full historic MaxEnt model with 100 background points. The limiting factor at background points (black) and occurrences (white) is enlarged around the occurrences.

There were shifts in the environmental parameters related to gopher occurrences from the 1950s to the contemporary era. Contemporary occurrences had lower elevations (*p* < .001), higher mean precipitation (*p* < .001), higher standard deviation of precipitation (*p* < .001), and sandier soil textures (*p* < .001), and there was a shift from agricultural areas toward areas dominated by mild development (*p* < .001; Figure 4). Historic occurrences from 1945 to 1955 (*n* = 82) and 1956 to 1981 (*n* = 45) were predominantly located in agriculture present day (80%, Figure C4 in Appendix C) and were not occupied during resurveys in 2017–2018. From the limiting factors analysis, land cover was the primary limiting factor at contemporary gopher occurrences, and mean precipitation was the primary limiting factor at historic occurrences (Figures E1 and E2 in Appendix E).

**FIGURE 5:**
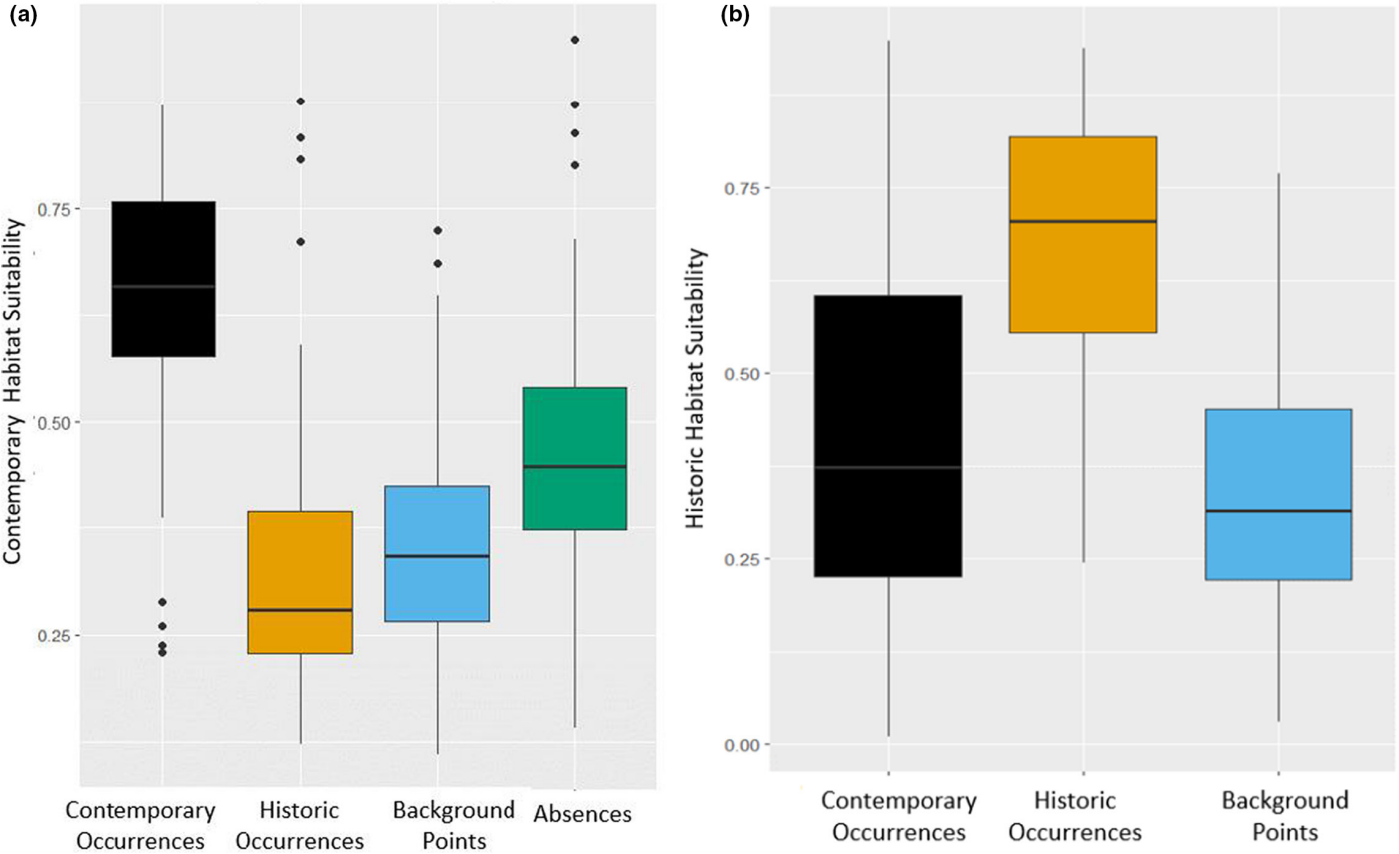
Predicted habitat suitability for pocket gophers in Illinois from the (a) full contemporary MaxEnt model and (b) full historic MaxEnt model (with both using 100 background points) at contemporary occurrences, historic occurrences, background locations, and contemporary absences.

Model transferability was low between the historic full model and the contemporary full model with 100 background points (Figure 5). The historic model estimated contemporary gopher occurrences as having only slightly higher habitat values than background points, and the contemporary model estimated historic occurrences as having lower habitat suitability than background points and contemporary absences. The models were unable to predict suitable occurrences between eras and therefore were not transferable.

### 3.4 Niche hypervolumes

**FIGURE F1:**
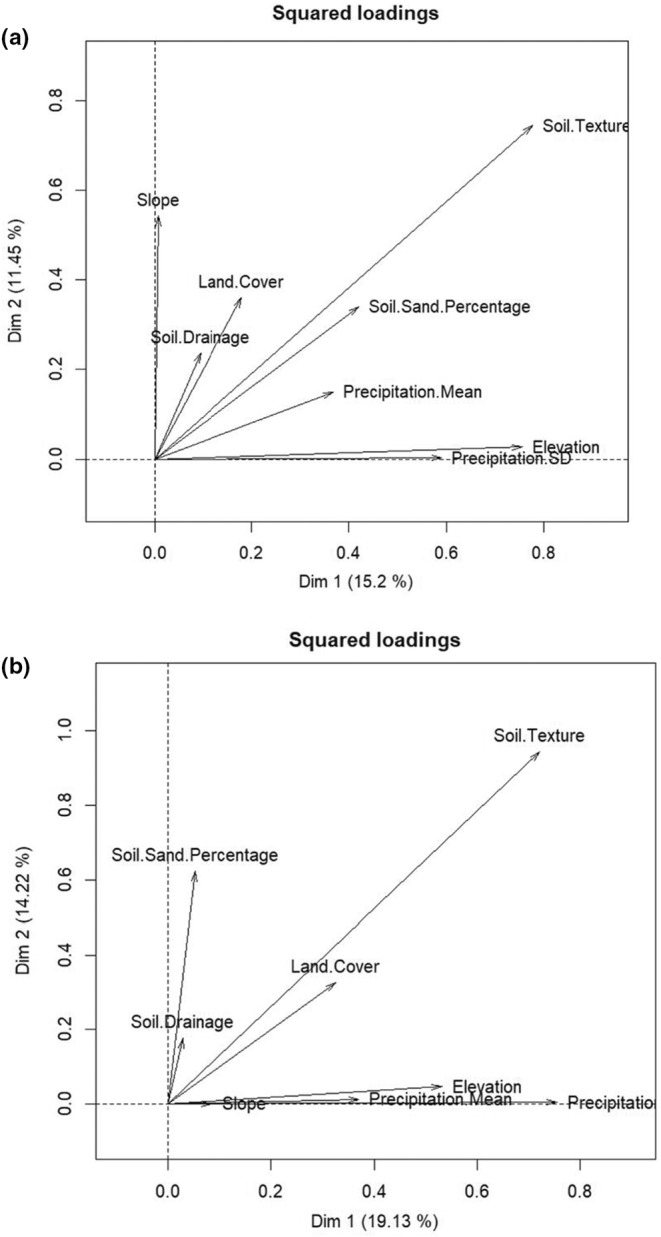
Squared loadings of parameter contributions to the PCA used in estimating niche hypervolume for pocket gophers in Illinois based on (a) contemporary and (b) historic occurrences.

**FIGURE F2:**
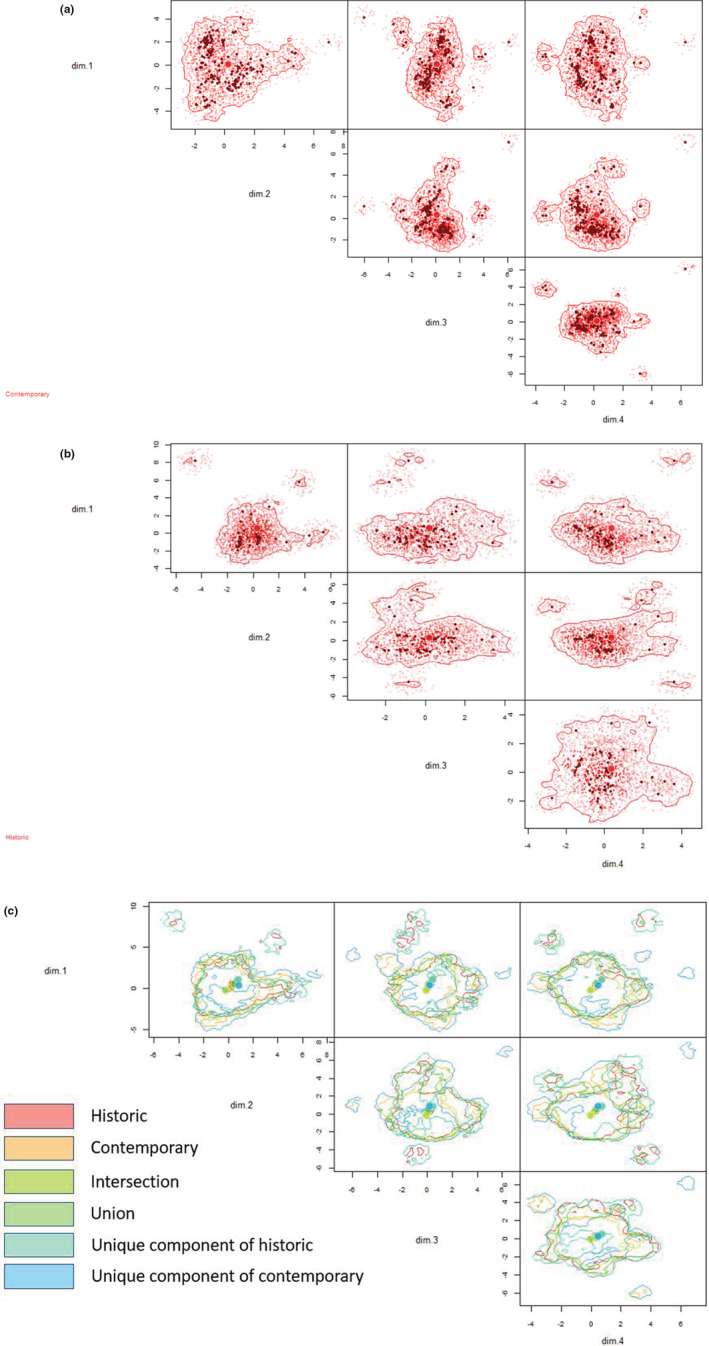
Two‐dimensional hypervolume plots of the first four PCA axes for (a) contemporary and (b) historic niche hypervolumes for pocket gophers in Illinois and (c) the contemporary niche hypervolume compared with the historic niche hypervolume along with their intersection, union, and their respective unique niche hypervolumes for the first four PCA axes.

The first four dimensions of the PCAMix explained 41.9% of the variation of the environmental parameters at contemporary gopher occurrences and 53.7% of the variation for the historic occurrences. Both the contemporary and historic niche models identified soil texture as a large contributor to the PCA from the squared loadings (Figure F1 in Appendix F). We documented a 16.8% decrease in niche volume between eras (historic volume = 661, contemporary volume = 550) with a centroid distance of 0.482 and a Sørensen similarity of 0.483. Overall, the contemporary niche hypervolume has shifted from the historic niche, and the two niche hypervolumes were only 48.2% similar. We also observed holes within the hypervolumes (Figure F2 in Appendix F). Niche hypervolume differences were due to both a niche reduction and a niche shift.

## 4 DISCUSSION

Our study indicates gophers in Illinois have experienced niche shifts and a niche reduction over time as evidenced by changes in limiting factors, shifts in environmental associations, and a reduced niche hypervolume. By testing the niche reduction hypothesis, we were able to understand how nonuniform spatial responses to threats can underlie a species' decline and range contraction (Scheele et al., 2017). We observed reduced occurrences of gophers in the interior of their range, similar to patterns for other declining species (Lomolino & Channell, 1995). We predicted that gophers would be associated with grasslands, sandy‐clay loam soils, high elevation, low precipitation, and high soil drainage, with reductions in agricultural and grassland suitability over time. Overall, our results supported these predictions, but with further complexities. We start by discussing the documented niche reduction and shift, changes in model contributions and limiting factors for gopher habitat suitability, and the altered environmental niche associations. Next, we discuss potential species‐specific concerns for these niche shifts. We conclude with how the niche reduction hypothesis can be applied more broadly, specifically in other systems with agricultural intensification.

### 4.1 Niche reduction and shifts

We observed imperfect spatial niche overlap and a reduced niche hypervolume between the historic and contemporary eras. From the SDMs, we documented a shift in environmental drivers from elevation, precipitation, and land cover to land cover, soil texture, and soil drainage based on parameters included in the selected models and the results from the jackknife analyses. The main limiting factor across the gopher range shifted from being mean precipitation to predominantly being land cover. The change from associations with high elevation and precipitation to associations with soil texture and drainage suggests soil water retention and burrow flooding may be important constraints on gopher distribution. However, increasing precipitation regimes and farm expansion into areas of higher elevation have altered how gophers avoid flooded burrows. Gophers create vertical burrows, likely for drainage, but increased flooding may prevent population establishment or persistence (Davis et al., 1938). Flooding can collapse burrows and force gophers to abandon them (Miller, 1957; Thorne & Andersen, 1990), decrease burrow excavation rates (Mohr & Mohr, 1936), or drown gophers (Kershen, 2004). Overall, water dynamics may impact the gopher niche more than previously thought due to increasing precipitation regimes and agricultural development in areas with adequate drainage.

Although the niche reduction hypothesis can apply to prey responding to biotic interactions including predation (e.g., McDonald et al., 2018), there is limited information on predation for gophers (e.g., Connior & Risch, 2010). There may be a wide array of opportunistic predators of gophers; however, American badgers (*Taxidea taxus*) are uniquely able to excavate gopher burrows (Hoffmeister, 1989). Monitoring data are lacking to establish whether the distribution or abundance of badgers has changed from historical to contemporary eras in Illinois. However, badgers are a prairie obligate species with unusually large home ranges in the current fragmented landscape (Duquette et al., 2014) and likely have declined over time. During our presence–absence surveys, we detected a single badger burrow, and during our driving surveys, we detected a single road‐killed badger. As gophers have shifted to mildly developed areas, cats (*Felis catus*) may increasingly pose predation risks to gophers.

### 4.2 Species‐specific concerns

Crops may have similar restrictions as gophers do in terms of the water table. For example, corn yield relationships vary with the shallow water table (saturated soil within top 2 m of soil profile) in which too much water near the surface may damage crops, even though water at lower soil levels may provide stability during drought (Rizzo et al., 2018). Farmers view land as marginal partially based on likelihood of flooding (Yang et al., 2021), and with increasing precipitation regimes, farmers may view higher elevation as more optimal. This would displace gophers from higher elevations, leading gophers to occupy lower elevations with sandier soils to maintain burrow drainage. Tile drainage, a practice to remove excess subsurface water from croplands, can alleviate flooding, but most of the drainage is old, implemented for crops with high transpiration (e.g., alfalfa transpires more than soybeans), and is not adequate for increased precipitation from climate change (Castellano et al., 2019). Tile drainage is also not commonly used in the western, sandier areas of the gopher distribution in Illinois, but is used frequently in the northeast region of the gopher range (Rizzo et al., 2018). Recent mapping of tile drainage using geospatial modeling (Valayamkunnath et al., 2020) could increase our understanding of subterranean species distributions in relation to agricultural water management, especially as drainage systems are updated to meet agricultural needs.

Increased precipitation, including more extreme events, from climate change may exclude species from historically occupied areas (Widick & Bean, 2019) or extirpate entire populations (Mason‐Romo et al., 2018; McCain & Colwell, 2011; Wilkening et al., 2019). We observed an increase in precipitation between eras across the background, historic, and contemporary locations, likely due to climate change. Gophers used to persist at the upper limits of precipitation regimes sampled historically (~980 mm), but they now persist at the lower limits of current precipitation regimes (~1020 mm; Figure 4). Future increases in precipitation in the region could further decrease the realized niche for gophers. Interactions between climate change and land cover also may exacerbate the risk for species (Williams et al., 2021).

Agriculture has also expanded into more poor‐quality lands due to increasing corn prices (Aragon, 2019) and has brought conservation lands into use (Holland et al., 2020; Morefield et al., 2016). Soil loss in agricultural systems leads to reduced crop yield and income (Thaler et al., 2021), further increasing the intensification and production on erodible land (Holland et al., 2020). As agricultural practices fluctuate based on economic return, farm expansion and land use may further restrict gopher suitability as soils are eroded from previously suitable areas or poor‐quality soils are brought into production.

Farm expansion into marginal lands, soil erosion, and changes in agricultural production due to precipitation all may displace gophers, but we observed additional declines in agricultural suitability over time. This reduction of habitat suitability, and occurrences of gophers within agricultural lands, indicates a decrease in niche space similar to other grassland rodents (Nikolić et al., 2019; Rutrough et al., 2019). Since the 1950s, historic sites have remained in agricultural production but had no contemporary gopher occurrences, indicating reduced suitability. Gophers can occupy alfalfa (Hoffman & Choate, 2008) and hayfields (Sietman et al., 1994), but alfalfa and hay production in Illinois has declined, replaced with corn and soybeans (Garcia et al., 1987; Sulc & Tracy, 2007; Warner, 1994). Hence, this decline in habitat suitability for gophers coincides with agricultural practices that increased annual row crops, increased farm size with loss of grassland buffers, and increased soil compaction from heavier machinery (Keller & Or, 2022).

### 4.3 Broader applications

Although habitat loss and fragmentation are of conservation concern due to isolating populations and decreasing genetic diversity, how those habitat changes occur can impact species‐specific adaptive potential and conservation outcomes. Gophers have strong associations with soil classes (Connior, 2011; Connior et al., 2010; Hoffman & Choate, 2008; Warren et al., 2017) including creating boundaries between gopher species and subspecies (Connior, 2011; Genoways et al., 2008; Henke et al., 2014). Given that gophers in Illinois are becoming increasingly restricted to certain soil properties, genetic‐soil associations may be lost if populations within unique soils are extirpated. Conservation efforts should focus on both gopher persistence in the contemporary soil associations as well as re‐establishment within historically occupied soils.

The niche reduction hypothesis can inform conservation recommendations based on historic and contemporary realized niches (Scheele et al., 2017). By identifying niche space that was historically occupied concurrently with the contemporary niche, managers can attempt to restore habitat lost from the historic niche while implementing efforts to conserve existing habitat. For gophers, areas of higher elevation and with silty loam soils can be prioritized for genetic conservation, but areas of high gopher occurrence, such as areas with high sand percentage or in sandy loam soils, could also be prioritized for population conservation.

The niche reduction hypothesis also frames practices of conservation concern as dynamic and is useful for landscapes continuing to change after initial conversion to agriculture. We recommend applying the concept to other systems undergoing agricultural intensification and becoming monocultures (Roesch‐McNally et al., 2018). The U.S. Corn Belt has brought more land into production as well as increased yield, but crop type is heavily dependent on economic incentives and crop prices, creating temporally changing landscapes and conservation practices (Lin & Huang, 2019). With further studies on niche reduction of species in agriculturally intensifying systems, dynamic strategies incorporating wildlife conservation, sustainable agricultural management, and economics can be developed to meet the complex goals of agroecosystems (Hunter et al., 2017).

## AUTHOR CONTRIBUTIONS

**Nathan Alexander:** Conceptualization (equal); data curation (lead); formal analysis (lead); investigation (lead); methodology (lead); writing – original draft (lead); writing – review and editing (lead). **Bradley J. Cosentino:** Conceptualization (supporting); formal analysis (supporting); funding acquisition (equal); investigation (supporting); methodology (supporting); project administration (supporting); resources (equal); writing – review and editing (supporting). **Robert L. Schooley:** Conceptualization (equal); formal analysis (supporting); funding acquisition (equal); investigation (supporting); methodology (supporting); project administration (lead); supervision (supporting); writing – review and editing (supporting).

## CONFLICT OF INTEREST

The authors declare that they have no competing interests in relation to the publication of this paper.

## Data Availability

Data available on Dryad, Dataset (Alexander et al., 2022).

## APPENDIX A

### Soil texture and soil drainage classifications within the range of *Geomys bursarius*

**TABLE A1:** Numeric code for soil texture within the range of *Geomys bursarius* in Illinois.

Numeric code	Soil texture classification
1	Fine sand
2	Clay loam
4	Fine sandy loam
5	Stratified sandy loam to silt loam
6	Silty clay loam
7	Silt loam
8	Sandy loam
11	Stratified sand to loam
12	Silty clay
13	Loam
14	Very cobbly silt loam
16	Stratified gravelly sandy loam to silt loam
17	Very channery loam
18	Very fine sandy loam
19	Stratified sand to clay loam
20	Gravelly coarse sand
22	Stratified very fine sandy loam to silty clay loam
26	Variable

**TABLE A2:** Numeric code for soil drainage within the range of *Geomys bursarius* in Illinois.

Numeric code	Soil drainage classification
2	Somewhat excessively drained
3	Well drained
4	Moderately well drained
5	Somewhat poorly drained
6	Poorly drained
7	Very poorly drained

**TABLE A3:** Numeric code, the land cover classification from the United States Department of Agriculture National Agricultural Statistics Service (2019), and the reassignment land cover classification for the reduced land cover within the range of *Geomys bursarius* in Illinois.

Numeric code	Land cover	Assigned reduced land cover
1	Corn	Agriculture
2	Cotton	Agriculture
3	Rice	Agriculture
4	Sorghum	Agriculture
5	Soybeans	Agriculture
12	Sweet corn	Agriculture
13	Pop or orn corn	Agriculture
21	Barley	Agriculture
23	Spring wheat	Agriculture
24	Winter wheat	Agriculture
26	Double‐crop winter wheat/soybeans	Agriculture
27	Rye	Agriculture
28	Oats	Agriculture
29	Millet	Agriculture
36	Alfalfa	Agriculture
37	Other hay/non‐alfalfa	Agriculture
42	Dry beans	Agriculture
43	Potatoes	Agriculture
44	Other crops	Agriculture
47	Miscellaneous vegetables & fruits	Agriculture
48	Watermelons	Agriculture
49	Onions	Agriculture
53	Peas	Agriculture
54	Tomatoes	Agriculture
57	Herbs	Agriculture
58	Clover/wildflowers	Grassland/pasture
59	Sod/grass seed	Grassland/pasture
60	Switchgrass	Grassland/pasture
61	Fallow/idle cropland	Grassland/pasture
67	Peaches	Orchard
68	Apples	Orchard
70	Christmas trees	Orchard
71	Other tree crops	Orchard
74	Pecans	Orchard
76	Walnuts	Orchard
77	Pears	Orchard
111	Open water	Water
121	Developed/open space	Grassland/pasture
122	Developed/low intensity	Dev Low
123	Developed/med intensity	Dev Med
124	Developed/high intensity	Dev High
131	Barren	grassland/pasture
141	Deciduous forest	Forest
142	Evergreen forest	Forest
143	Mixed forest	Forest
152	Shrubland	Forest
176	Grassland/pasture	Grassland/pasture
190	Woody wetlands	Forest
195	Herbaceous wetlands	Forest
205	Triticale	Agriculture
206	Carrots	Agriculture
216	Peppers	Agriculture
222	Squash	Agriculture
225	Double‐crop winter wheat/corn	Agriculture
229	Pumpkins	Agriculture
236	Double‐crop winter wheat/sorghum	Agriculture
237	Double‐crop barley/corn	Agriculture
240	Double‐crop soybeans/oats	Agriculture
242	Blueberries	Agriculture
249	Gourds	Agriculture
254	Double‐crop barley/soybeans	Agriculture

## APPENDIX B

Area under the curve (AUC), parameter contributions, and jackknife analysis for the selected contemporary model, the selected contemporary model with reduced land cover classification, and the selected historic model

We did model selection for a suite of models with 63 classifications of land cover to determine whether specific crop types were worse for gophers and then did model selection using a reduced land cover classification (*n* = 7) to ensure we did not overparameterize the model. Both contemporary models identified the same parameters and had similar response curves and jackknife analyses. The two contemporary models had a spatial niche overlap (Warren's *I*) of 0.99; thus, we consider the model with the full land cover classifications to be comparable to a model with fewer land cover classifications.

## APPENDIX C

Field surveys including validation of contemporary habitat suitability model including the resurvey points (C1) random‐forest classification tree (C2), accuracy and impact on the Gini index (C3), and contemporary surveys at historic occurrence locations (C4)

## APPENDIX D

Spatial niche overlap between the historic and contemporary best and global models as well as the impact of the number of background points in a MaxEnt model

To quantify niche overlap for the best contemporary and historic SDMs, we also compared Warren's *I* between the top models. We also wanted to account for the effect of sample size in niche overlap because MaxEnt is sensitive to the number of background points used. Hence, we estimated Warren's *I* between (1) the contemporary full model with 10,000 random background points and reduced land cover categories, (2) the contemporary full model with the specified 100 background points and reduced land cover categories, (3) the selected contemporary model with 10,000 background points and all land cover categories, (4) the historic full model with the specified 100 background points and reduced land cover categories, and (5) the selected historic model with the specified 100 background points and reduced land cover categories.

To determine the impact of the number of background points on our models and the impact of using full models rather than top performing models, we used Warren's *I* to estimate spatial niche overlap. The contemporary full model with 10,000 background points varied the most with the contemporary full model with 100 background points and the selected contemporary model. Because Warren's *I* estimates spatial overlap of niche, contemporary models using the full extent have a larger area of predicted SDM values to compare, whereas the historic SDMs are constrained to the 1km buffer around predicted areas we were able to digitize; thus, the contemporary models may be expected to have higher variation. Of note, the selected contemporary and the selected historic models only had a Warren's *I* of 0.791.

**TABLE D1:** Warren's *I* index, which ranges from 0 to 1 and measures niche similarity, for the full contemporary model, the top selected contemporary model, the full historic model, and the top selected historic model.

	Contemporary full^a^	Contemporary full^b^	Selected contemporary^a^	Historic full^b^
Contemporary full^a^	0			
Contemporary full^b^	0.897	0		
Selected contemporary^a^	0.953	0.874	0	
Historic full^b^	0.763	0.920	0.757	0
Historic selected^b^	0.760	0.950	0.791	0.981

*Note*: Similarity was quantified for models with all contemporary land cover categories and 10,000 background points (a) and with reduced land cover categories and 100 background points (b).

## APPENDIX E

MaxEnt limiting factor analysis for the contemporary full MaxEnt model of *Geomys bursarius* (10,000 background points) and the historic full MaxEnt model (100 background points). The limiting factor analysis identifies which factor limits a MaxEnt model within each raster cell

## APPENDIX F

Principal component analysis (PCA) loadings and Niche hypervolume plots
